# Identification of environmental chemicals that activate p53 signaling after in vitro metabolic activation

**DOI:** 10.1007/s00204-022-03291-5

**Published:** 2022-04-18

**Authors:** Masato Ooka, Jinghua Zhao, Pranav Shah, Jameson Travers, Carleen Klumpp-Thomas, Xin Xu, Ruili Huang, Stephen Ferguson, Kristine L. Witt, Stephanie L. Smith-Roe, Anton Simeonov, Menghang Xia

**Affiliations:** 1 National Center for Advancing Translational Sciences, National Institutes of Health, 9800 Medical Center Drive, Rockville, MD 20850 USA; 2 Division of the National Toxicology Program, National Institute of Environmental Health Sciences, National Institutes of Health, Research Triangle Park, NC 27709 USA

**Keywords:** In vitro metabolism, High-throughput screening, Microsomes, p53, High-throughput metabolism assay, In silico metabolism prediction

## Abstract

Currently, approximately 80,000 chemicals are used in commerce. Most have little-to-no toxicity information. The U.S. Toxicology in the 21st Century (Tox21) program has conducted a battery of in vitro assays using a quantitative high-throughput screening (qHTS) platform to gain toxicity information on environmental chemicals. Due to technical challenges, standard methods for providing xenobiotic metabolism could not be applied to qHTS assays. To address this limitation, we screened the Tox21 10,000-compound (10K) library, with concentrations ranging from 2.8 nM to 92 µM, using a p53 beta-lactamase reporter gene assay (p53-bla) alone or with rat liver microsomes (RLM) or human liver microsomes (HLM) supplemented with NADPH, to identify compounds that induce p53 signaling after biotransformation. Two hundred and seventy-eight compounds were identified as active under any of these three conditions. Of these 278 compounds, 73 gave more potent responses in the p53-bla assay with RLM, and 2 were more potent in the p53-bla assay with HLM compared with the responses they generated in the p53-bla assay without microsomes. To confirm the role of metabolism in the differential responses, we re-tested these 75 compounds in the absence of NADPH or with heat-attenuated microsomes. Forty-four compounds treated with RLM, but none with HLM, became less potent under these conditions, confirming the role of RLM in metabolic activation. Further evidence of biotransformation was obtained by measuring the half-life of the parent compounds in the presence of microsomes. Together, the data support the use of RLM in qHTS for identifying chemicals requiring biotransformation to induce biological responses.

**Supplementary Information:**

The online version contains supplementary material available at 10.1007/s00204-022-03291-5.

## Introduction

An increasing number of chemicals continues to be produced and released into the environment. To evaluate the potential effects of these chemicals quickly and efficiently, the U.S. government established the Tox21 program, a multiagency effort that includes the National Center for Advancing Translational Sciences, National Institute of Environmental Health Sciences, Environmental Protection Agency, and Food and Drug Administration. (Kavlock et al. 2009). This program has utilized a quantitative high-throughput screening (qHTS) approach to assess a 10,000-compound (10K) library using a group of biologically and toxicologically relevant in vitro assays (Huang et al. 2016; Xia et al. 2018). Since 2008, more than 70 qHTS assays have been developed (Huang et al. 2016; Lynch et al. 2020), including cell- and enzyme-based assays. Although these assays can detect chemical effects on many different biological endpoints, most of them cannot detect compounds that require metabolism for activity (Witt et al. 2017) because the cell lines used in these assays do not have sufficient metabolic capability to biotransform parent compounds to active metabolites (Qu et al. 2021). Metabolism can activate or deactivate a parent compound. Since most compounds, including drugs, food additives, agrochemicals, and other environmental chemicals, are metabolized in humans, incorporating metabolism methods in qHTS assays is essential for assessing the full range of effects of compound exposure (Ooka et al. 2020).

Several methods for providing metabolic activation capability to the Tox21 qHTS assays were considered. In standard, low-throughput in vitro toxicity assays, induced rat liver S9 or microsomes are frequently used to provide xenobiotic metabolism. Although induced rat liver S9 fraction is highly effective at metabolizing compounds in cell-based assays, it is cytotoxic and must be removed from cell cultures after a few hours (Cox et al. 2016). This cytotoxicity has presented a difficult challenge for qHTS assays that use 1536-well plate formats, as wash steps are not generally compatible with these assay protocols. Thus, use of induced rat liver S9 has not generally been considered a viable option although a low concentration of S9 was recently shown to be effective in providing metabolic activation for an in vitro genotoxicity assay conducted using a 96-well plate format (Tian et al. 2020). In addition, the Alginate Immobilization of Metabolic Enzymes (AIME) hepatic metabolism method has also been used to provide metabolic capability to high-throughput in vitro screening platforms. The AIME method applies engineered lids with solid supports containing micro-spheres of hepatic S9 attached to an alginate matrix to induce metabolism in 96- or 384-well plate formats (Hopperstad et al. 2022). Another option for providing metabolic capability to in vitro assays is to co-culture the assay cells with human hepatocytes. However, although human hepatocytes closely mimic the metabolic capability of the human liver, they are difficult to work with and not well-suited to qHTS assays due to the cost and large lot-to-lot variation; they are better suited to low-throughput in vitro assays.

The activity of identified metabolites can be measured in enzyme-based assays, which have high reproducibility but do not authentically recapitulate a normal physiological environment. (Li et al. 2021). Therefore, translation of the results to human exposure scenarios is difficult. In the current study, we opted to use liver microsomes to provide metabolic capability to a p53-bla assay (Witt et al. 2017). Liver microsomes contain major Phase I metabolic enzymes such as cytochrome p450s (CYPs), which are essential to metabolism. Among other important functions, such as detoxifying xenobiotics, these CYPs are responsible for most of the metabolism of marketed drugs (Iyanagi 2007; Zanger et al. 2013). Microsome preparations do not contain some Phase II enzymes; therefore, they cannot mimic the full range of metabolism that occurs in the liver.

p53 is a tumor suppressor gene (Gnanapradeepan et al. 2018), working in a network of stress-response signaling pathways; p53 is most often activated in response to DNA damage and is therefore used as a biomarker for DNA damage (Kastenhuber et al. 2017; Krejci et al. 2008). In normal cells, the expression of p53 is maintained at low levels (Zhao et al. 2000). The p53-bla assay conducted in this investigation is a cell-based assay with high sensitivity and reproducibility; our laboratory has extensive experience conducting this assay (Witt et al., 2017). The HCT-116 cells used for the p53-bla assay do not have sufficient endogenous capability to metabolize parent compounds. Thus, this assay as originally conducted allowed us to identify activity associated only with the parent compounds; some compounds in the Tox21 library were known to induce DNA damage in standard in vitro assays that incorporated exogenous metabolic activation (induced rat liver S9), but they were not detected in our qHTS p53-bla assay.

Methods for providing exogenous metabolism capability have generally not been included in traditional HTS cell-based assays run in 1536-well plate formats because of concerns for cytotoxicity, reproducibility, and cost. In addition, the need for wash steps has also presented a hurdle (Ooka et al. 2020), since aspiration may create unacceptable inter-well variability in assay volumes. In this study, we used RLM and HLM with a p53-bla reporter gene assay to mimic biologically relevant conditions, since low amounts of microsomes retain their effectiveness, but have low levels of cytotoxicity. Therefore, this approach eliminates the requirement for a wash step. We used RLM and HLM to provide metabolism and compared the screening performances with and without microsomes. Assay reproducibility was evaluated, and compounds were ranked by potency and efficacy. We also investigated the depletion of the parent compounds under microsome treatment, confirming the biotransformation of the compounds.

## Materials and methods

### Reagents

CellSensor^™^ p53RE-bla HCT-116 cell line (p53-bla), which contains a beta-lactamase reporter gene under control of a stably integrated p53 response element for induction of p21 (*CDKN1A*) and cell culture reagents were obtained from Invitrogen (Life Technologies, Madison, WI). Mitomycin C, aflatoxin B1, and β-nicotinamide adenine dinucleotide 2′-phosphate (NADPH) were purchased from Sigma-Aldrich (St. Louis, MO).

### Cell culture

p53-bla cells were cultured in McCoy’s 5A medium supplemented with 10% dialyzed fetal bovine serum, 100 U/mL penicillin, 100 µg/mL streptomycin, and 5 µg/mL blasticidin. The cells were maintained at 37 °C under a humidified atmosphere and 5% CO_2_.

### p53 beta-lactamase reporter assays

p53-bla cells were resuspended in assay medium (Opti-MEM supplemented with 0.5% dialyzed fetal bovine serum) and dispensed at 4000 cells/3µL/well in 1,536-well black wall/clear bottom plates (Greiner Bio-One North America, Monroe, NC) using a Multidrop Combi (Thermo Fisher Scientific Inc., Waltham, MA). After incubation at 37 °C for 5 h, 23 nL of compound dissolved in dimethyl sulfoxide (DMSO) or DMSO only was added to the assay plates via Wako Pintool station (Wako Automation, San Diego, CA). For the assays with microsomes, 3 µL of Aroclor 1254-induced RLM (Molecular Toxicology, Boone, NC) or pooled donor HLM (XenoTech, Kansas City, KS) at a final concentration of 0.5 mg/mL and 1 µL of NADPH at final concentration 0.5 mg/mL were transferred to the assay plate by a Flying Reagent Dispenser (Aurora Discovery, Carlsbad, CA). Concentration range-finding studies were conducted to select microsomes and NADPH concentrations that did not induce notable cytotoxicity. To attenuate microsomes activities, the microsomes were pre-treated at 56 °C for 30 min. The assay plates were then incubated for an additional 16 h. On the next day, 1 µL of LiveBLAzer^™^ (Life Technologies, Madison, WI) detection mixture was added to each well and the plates were incubated at room temperature in the dark for 2 h. Fluorescence intensity at 460 and 530 nm emission and 405 nm excitation was measured by an Envision plate reader (PerkinElmer, Boston, MA). Data were represented as the ratio of the emission wavelengths (460/530). Next, 3 µL of CellTiter Glo reagent was added to each well and plates were incubated at room temperature in the dark for 30 min. Luminescence values were acquired on a ViewLux plate reader (Perkin Elmer) as a measure of cell viability. The activity of each test compound was measured at 15 concentrations in triplicate.

### The measurement of the depletion of parent compounds

The method we used for measuring the depletion of parent compounds was described previously (Shah et al. 2016). Briefly, each reaction mixture (110 μL) consisted of a test compound (1 μM), male Sprague Dawley rat microsomal fractions (0.5 mg/mL), and the NADPH regenerating system in phosphate buffer at pH 7.4. Reaction mixtures were incubated in 384-well plates at 37 °C for 0, 5, 10, 15, 30, and 60 min. Parent compounds were measured using mass spectrometry and half-life calculations of biotransformation time were performed using a previously described method (Shah et al. 2016). The three reference compounds used were buspirone (short half-life of 7.2 ± 2.3 min), loperamide (moderate half-life of 15.5 ± 2.1 min) and antipyrine (long half-life of > 120 min).

### In silico metabolism prediction

ADMET Predictor^®^ Version 8.5 (Simulation Plus, Lancaster, CA) was used for predicting parent compound metabolism. The metabolism modules of ADMET were run to evaluate the Michaelis–Menten constant (*K*_m_), maximum metabolic rate (*V*_max_), and intrinsic clearance (CL_int_) for the reference compounds. CL_int_ were calculated by *V*_max_/*K*_m_.

### Data analysis

Analysis of compound concentration–response data was performed as previously described (Huang 2016). Briefly, raw plate reads for each titration point were first normalized relative to the positive control compound (Mitomycin) and DMSO-only wells (0%) as follows:

$$\mathrm{\% Activity}= \frac{{V}_{\mathrm{compound}}-{V}_{\mathrm{neg}}}{{V}_{\mathrm{pos}}-{V}_{\mathrm{neg}}}\times 100,$$

where *V*_compound_ indicates the compound well values, *V*_pos_ indicates the median value of the positive control wells, and *V*_neg_ indicates the median values of the DMSO-only wells. The data set was then corrected using the DMSO-only compound plates at the beginning and end of the compound plate stack by applying an in-house pattern correction algorithm (Wang et al. 2016). The half maximum effective values (EC_50_) for each compound and maximum response (efficacy) values were obtained by fitting the concentration–response curves of each compound to a four-parameter Hill equation (Wang et al. 2010). Compounds were designated as class 1–4 according to the type of concentration–response curve observed (Huang 2016; Inglese et al. 2006). Curve classes were further combined with efficacy and converted to curve ranks, which are numeric measures of compound activity ranging from −9 to 9 (Huang 2016). Compounds that showed activation are assigned positive curve rank values and compounds that showed inhibition are assigned negative values. Inactive compounds are assigned a curve rank of 0. Each compound was assigned an activity outcome of inactive, active (agonist or antagonist), or inconclusive, based on the type of concentration–response curve and reproducibility (3 independent runs) as described previously (Huang 2016). The Tox21 10K library was evaluated for purity and the 278 compounds tested in the follow-up study met the acceptable chemical quality control criteria (https://tripod.nih.gov/tox/samples). Data were analyzed and depicted using OriginPro 2015 (OriginLab Corp., Northampton, MA) and GraphPad Prism 5 (GraphPad Software, Inc., La Jolla, CA).

The compounds in the Tox21 10K library were clustered by structural similarity (Leadscope^®^ fingerprints; Leadscope, Inc., Columbus, Ohio) using the self-organizing map (SOM) algorithm (Kohonen 2006). Each cluster was evaluated for the degree of enrichment with active agonists and the significance of the enrichment was determined by the Fisher’s exact test. In the follow-up study, EC_50_ and efficacy were used to compare the activity for each compound among 4 conditions: without microsomes, with microsomes plus NADPH, with heat-attenuated microsomes plus NADPH, and with microsomes but without NADPH. The significances of the EC_50_ and efficacy values among the 4 experimental conditions were evaluated by an ANOVA test.

## Results

### Assay development and performance

To identify compounds that induce p53 after metabolic activation, we modified the p53-bla assay to include the addition of HLM or RLM. To establish optimal concentrations of NADPH, cells were treated with aflatoxin B1 at concentrations ranging from 2 nM to 40 µM in the presence of HLM (0.5 mg/mL), or RLM (0.5 mg/mL) with or without NADPH (0.125, 0.25, and 0.5 mg/mL), a CYP450 enzyme co-factor. The EC_50_ values for aflatoxin B1 in the presence of 0.5 mg/mL HLM were 4.5, 5.3, and 3.0 μM at NADPH concentrations of 0.125, 0.25, and 0.5 mg/mL, respectively (Supplementary Figure. S1a), while the EC_50_ values for aflatoxin B1 in the presence of 0.5 mg/mL RLM were 1.5, 1.1, and 0.34 μM at NADPH concentrations of 0.125, 0.25, and 0.5 mg/mL, respectively (Supplementary Figure. S1b). Aflatoxin B1 showed higher activity in the p53-bla assay in presence of RLM than with HLM. The highest signal-to-background ratios were seen with 0.5 mg/mL NADPH; the ratios were 2.13- and 1.64-fold, with HLM and with RLM, respectively. Therefore, 0.5 mg/mL NADPH was chosen for compound screening.

The p53-bla assay was then used to screen the Tox21 10K compound library, which contains 8312 unique compounds, with or without RLM or HLM. To evaluate the performance of the qHTS assay in this primary screening, three parameters were calculated as described previously (Zhang et al. 1999). Assay performance statistics of signal-to-background ratio (S/B ratio) > 2, coefficient of variance (CV) ≤ 10%, and *Z *factor ≥ 0.5 are considered good quality for primary screening. Based on these criteria, the assays performed well for most primary screening (Table 1). We further evaluated reproducibility of the primary screening by assessing three independent screening runs. After the primary screening, each compound was classified into one of four categories based on reproducibility of activity: active match, inactive match, mismatch, or inconclusive. The mismatch rate of the Tox21 10K primary screening was low, with a value of 0.01% for p53-bla assay without microsomes and 0% for p53-bla assay with RLM or HLM, indicating a good assay performance.

**Table 1:** Performance of the p53-bla assay in the absence or presence of rat/human liver microsomes

	Without microsomes	Rat liver microsomes	Human liver microsomes
CV	9.63 ± 1.04	4.37 ± 1.33	4.11 ± 1.52
Signal/background	3.45 ± 0.08	2.35 ± 0.09	1.53 ± 0.05
Z’ factor	0.74 ± 0.03	0.74 ± 0.07	0.48 ± 0.13

### Structure–activity relationships (SAR) of p53 activators that require metabolism

**Fig. 1:**
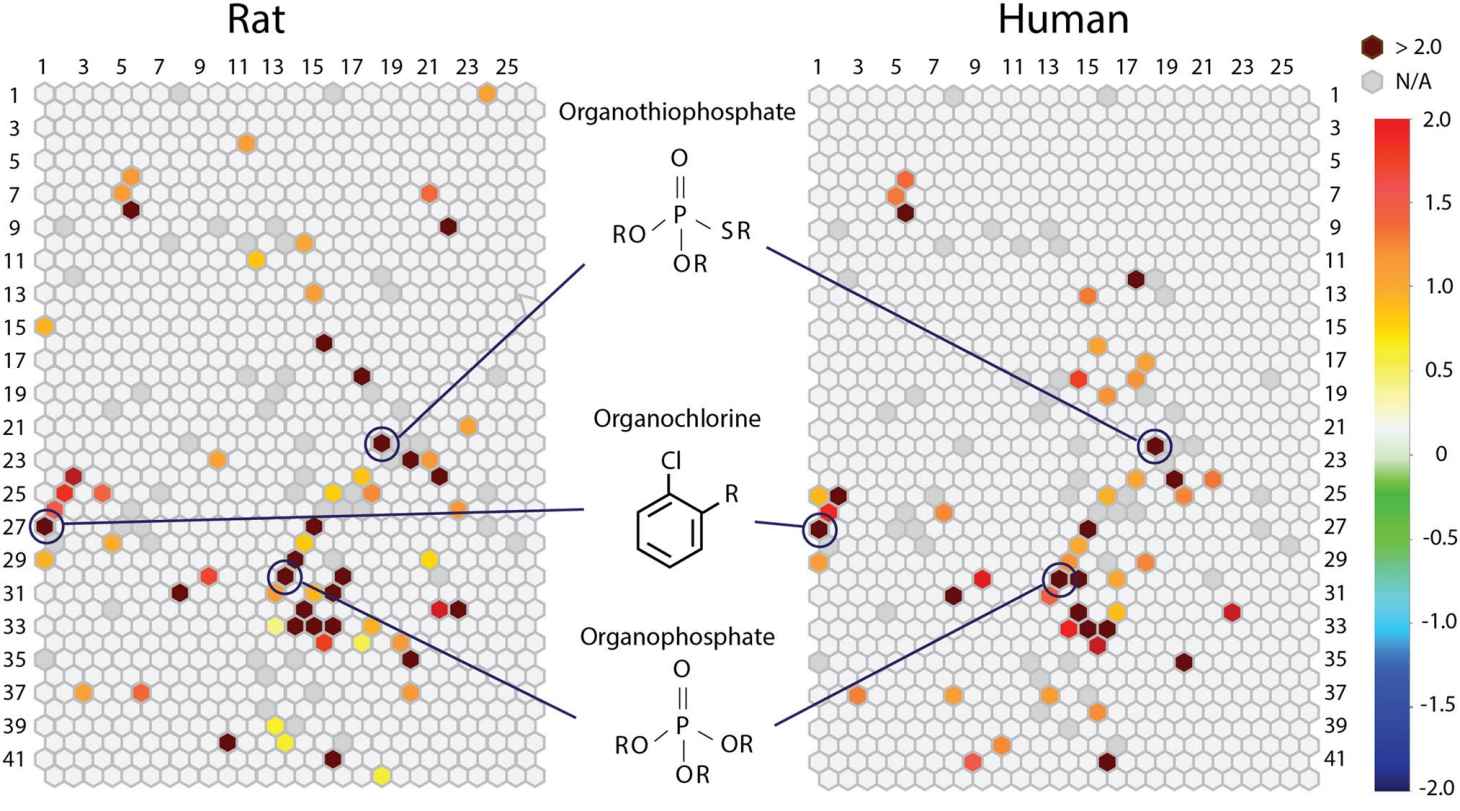
Structure–Activity Relationship (SAR) of compounds that induce the p53 signaling pathway. All compounds with associated structures present in the library were clustered, based on structural similarity using the Self-Organizing Map (SOM) algorithm (Kohonen 2006). Then, each cluster was evaluated for enrichment with active agonists (compared to the library average) using the Fisher’s exact test. In the heatmaps, each hexagon represents a cluster of structurally similar compounds. Clusters are colored by the significance of enrichment (negative log *p* value) such that more significantly enriched clusters are colored closer to a maroon color and the other clusters are colored with different shades as indicated in the color bar. A light gray color shade represents that the degree of enrichment of the cluster is close to the library average

The compounds in the Tox21 10K library were grouped into 1,014 clusters based on structural similarity (see methods for details). Each cluster was then examined for enrichment of p53 activators (compared to the library average) with RLM (Fig. 1, left panel) or HLM (Fig. 1, right panel). Twenty-two of the p53-bla plus RLM and fifteen of the p53-bla plus HLM structural clusters were significantly (*p* < 0.01) enriched with p53 activators (Fig. 1). Enriched clusters containing organophosphates were seen for both the p53-bla plus HLM and p53-bla plus RLM assays (Fig. 1, row 30, column 13). Organothiophosphates or organochlorines were also found in clusters enriched with p53 activators (Fig. 1, row 22, column 18, and row 27, column1). These classes of compounds are often used as pesticides (Karami-Mohajeri et al. 2011).

### Confirmation of selected potential p53 inducers

**Fig. 2:**
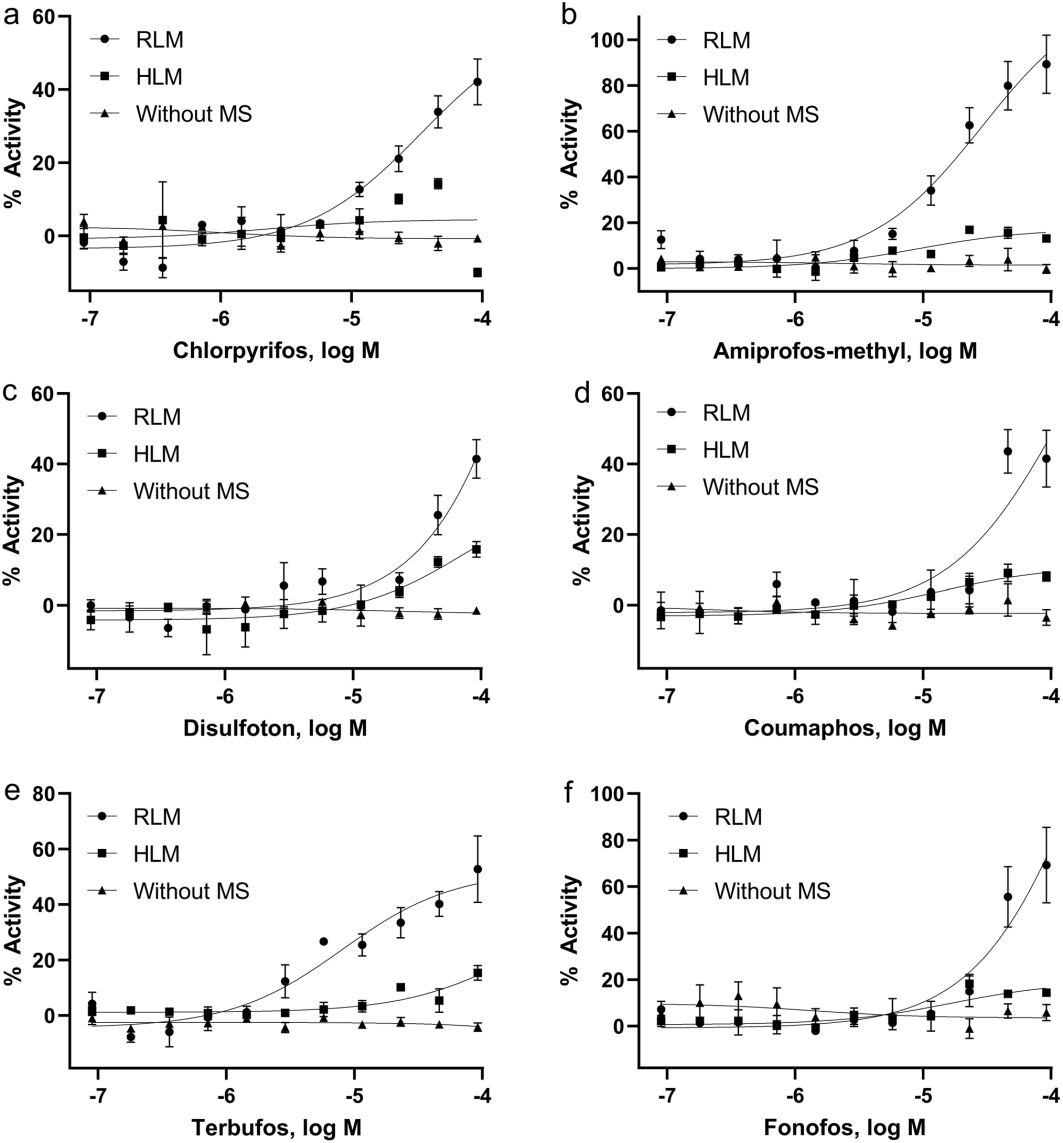
Concentration–response curves of representative compounds in p53-bla assays. Cells were treated with the compounds in the absence or presence of rat/human liver microsomes. **a** chlorpyrifos, **b **amiprofos-methyl, **c** disulfoton, **d** coumaphos, **e** terbufos, and **f** fonofos. Each value represents the mean ± SD of three independent experiments. Without MS, without microsomes

The primary screening identified 278 compounds out of 8312 unique compounds that potentially activate the p53 signaling pathway in either of the three conditions (Supplementary Table S1): without microsomes, with RLM, and with HLM (Supplementary Figure. S2a). These 278 compounds were re-tested in the p53-bla assay under the three conditions described above (Supplementary Figure. S2b). The confirmation rates for the three assays were 89.30, 88.56 and 78.23% for p53-bla, p53-bla plus RLM, and p53-bla plus HLM, respectively. Eighty-eight compounds were active in p53-bla plus RLM; 17 of these 88 were also active in the absence of RLM and one was active with both RLM and HLM but not without microsomes. Of the 88 compounds, 73 were more potent and/or more efficient with RLM compared to the responses observed in the p53-bla assay without RLM. Similarly, of the 6 compounds active in the p53-bla plus HLM assay, disodium 4,4'-bis (2-sulfostyryl) biphenyl was also active in the absence of microsomes but were more potent with HLM. Dipyridamole was identified as positive in both p53-bla plus RLM and p53-bla plus HLM, but not in p53-bla assay without microsomes. Using RLM, we were able to identify chlorpyrifos as an activator of p53 signaling; chlorpyrifos was inactive in the p53 assay without microsomes. These results are consistent with the fact that chlorpyrifos is known to be metabolized to a compound that induces DNA damage, a major cause of p53 activation (Fig. 2a) (Foxenberg et al. 2007; Wang et al. 2014). Using RLM, we also identified compounds not previously reported to induce p53 signaling, such as amiprofos-methyl, disulfoton, coumaphos, terbufos, and fonofos (Fig. 2b–f).

### Confirmation study with heat-attenuated microsomes

**Fig. 3:**
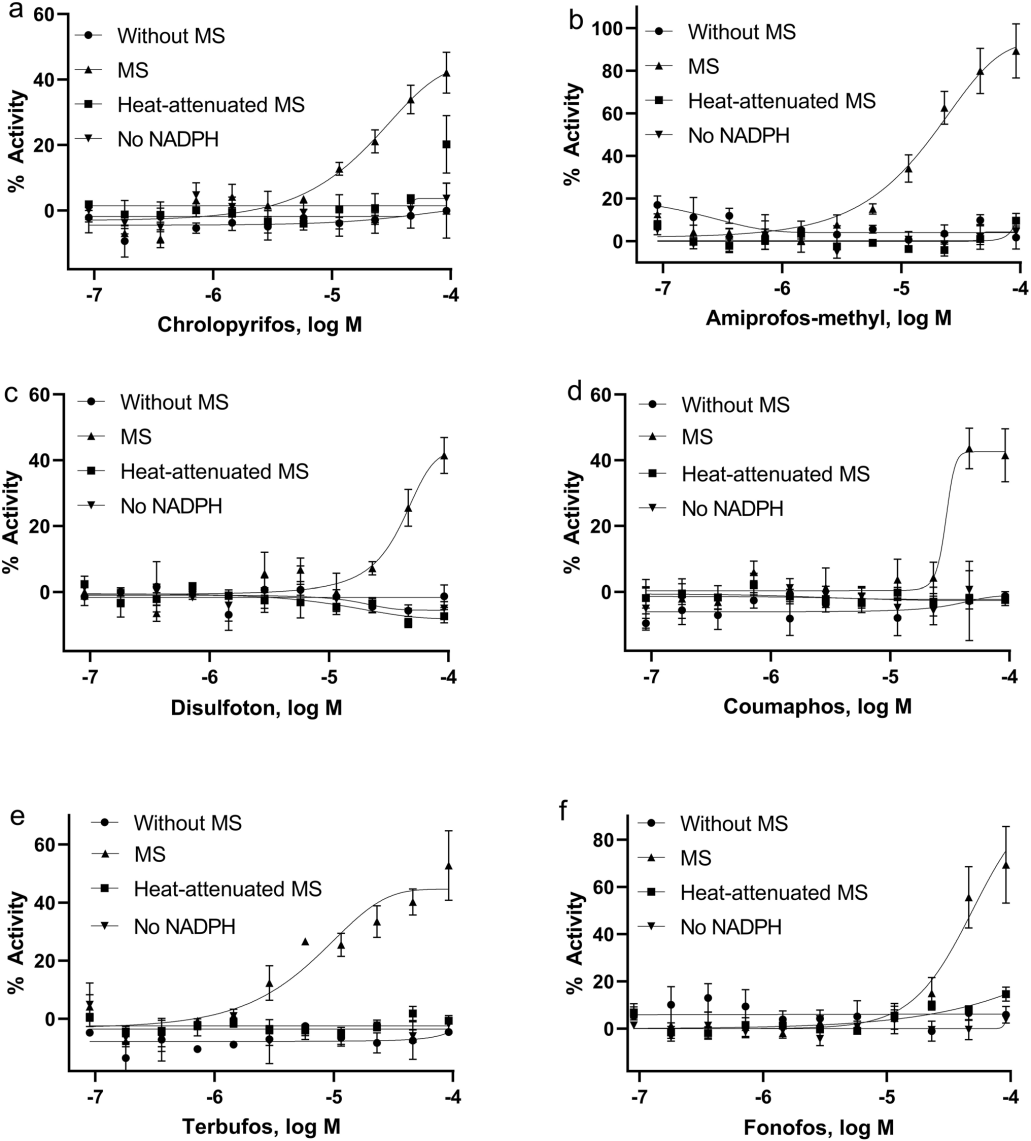
Concentration–response curves of representative compounds in p53-bla assays. Cells were treated with the compounds in the absence or presence of heat-attenuated rat liver microsomes or rat liver microsomes in the absence of NADPH. **a** chlorpyrifos, **b** amiprofos-methyl, **c** disulfoton, **d** coumaphos, **e** terbufos, and **f** fonofos. Each value represents the mean ± SD of three independent experiments. MS, with microsomes; Heat-attenuated MS, with heat-attenuated microsomes; No NADPH, without NADPH

To confirm that the activity of these p53 inducers was dependent on biotransformation, we tested these compounds in the p53-bla assay with heat-attenuated RLM or HLM, as well as without NADPH. Among the 73 compounds that showed higher responses, based on either potency or efficacy, for p53 induction with RLM compared to the assay without RLM, 44 compounds showed reduced activity with heat-attenuated RLM or no NADPH, yielding a confirmation rate of 60% (Supplementary Table S2). Because heat attenuation does not completely eliminate microsome activity, nor does the absence of NADPH, we did not expect to see reduced activity for all 73 compounds. These data suggest that the metabolites of these 44 compounds are more potent and/or more efficient inducers of p53 than the parent compounds. This group of 44 compounds included several phosphorylated compounds: chlorpyrifos (Fig. 3a), amiprofos-methyl (Fig. 3b), disulfoton (Fig. 3c), coumaphos (Fig. 3d), terbufos (Fig. 3e), and fonofos (Fig. 3f). Metabolism studies previously conducted with phosphorylated compounds demonstrated that their bioactivation is dependent on cytochrome P450 metabolism (Buratti et al. 2003; Foxenberg et al. 2007; Sams et al. 2004). The 2 compounds that were identified as more potent inducers of p53 with HLM did not show a significant decrease in potency when treated with heat-attenuated HLM.

### Measurement of parent compound depletion under microsome treatment

In this study, we observed that the p53-bla assay with RLM identified more actives than the p53-bla assay with HLM. To provide further evidence of the metabolic activation of these compounds, we performed compound stability experiments in the presence of RLM in a cell-free system (Shah et al. 2016). For this study, we focused on the subset of 44 compounds except leptophos (not commercially available) that showed higher potencies and/or efficacies when treated with RLM. These compounds were treated with RLM, and the depletion of the parent compounds was measured at 0, 5, 10, 15, 30, and 60 min using mass spectrometry. Parent compound half-lives (*t*_1/2_) were calculated for each compound and 120 min was set as the maximum time for significant parent compound depletion resulting from metabolism. Of the 43 compounds, 30 were metabolized (*t*_1/2_ < 120 min), 8 were undetectable, and 5 were found to be stable (*t*_1/2_ > 120 min) (Table 2). The inability of mass spectrometry to detect 8 of the compounds may have been due to compound insolubility in the selected solvent, non-specific compound binding, or weak mass spectrometry signal. Next, for these same 43 compounds, we again used mass spectrometry to measure the percentage of parent compound depletion in the cell-based p53-bla assay with RLM after the 16 h incubation period. Thirty-seven compounds were found to be depleted, and 4 were undetectable in the assay system. We also found that 2 compounds, cridanimod and irinotecan were stable in this assay.

**Table 2:** The stability of parent compounds in the presence of rat liver microsomes with or without cells

Compound	Microsome stability Half-life without cells [min]	Depletion after 16 h of incubation with cells [%]
Amiprofos-methyl	1.11	99.99 ± 0
Azinphos-methyl	1.32 ± 0.64	94.04 ± 0.16
Benzo(b)fluoranthene	Not found	Not found
Bromofos	Not found	Not found
Bromophos-ethyl	> 120	Not found
Carbophenothion	Not found	88.95 ± 4.15
Chlorfenvinphos	28.59 ± 1.85	55.04 ± 4.69
Chlorpyrifos	1.11 ± 0.07	92.74 ± 1.38
Coumaphos	0.88 ± 0.12	99.03 ± 0.34
Cridanimod	> 120	Not metabolized
Dipyridamole	7.32 ± 0.59	92.37 ± 0.54
Disulfoton	> 120	93.89 ± 4.12
EPN	4.45 ± 1.72	88.15 ± 1.39
Famphur	10.8 ± 1.96	87.7 ± 3.02
Fenamiphos	2.56 ± 0.11	99.41 ± 0.07
Fensulfothion	13.24 ± 1.25	84.02 ± 0.83
Fonofos	0.82 ± 0.25	97.33 ± 0.29
Fosthiazate	15.24 ± 1.93	71.2 ± 1.62
Iodenphos	Not found	99.99 ± 0
Irinotecan	> 120	Not metabolized
Isazofos	2.12 ± 0.14	97.88 ± 0.31
Isocarbophos	Not found	Not found
Isofenphos	1.25 ± 0.47	88.45 ± 1.49
Isoxathion	2.77 ± 0.25	94.65 ± 0.45
5-Methoxypsoralen	21.11 ± 3.72	45.7 ± 3.67
7-Methylbenzo(a)pyrene	Not found	52.01 ± 27.41
4-Methylumbelliferone	19.11	46.53 ± 5.89
4-Methylumbelliferone hydrate	26.74 ± 0.98	49.37 ± 6.23
Molinate	11.97 ± 0.18	97.58 ± 0.19
Norharman	Not found	85.03 ± 0.42
Parathion	Not found	98.86 ± 0.34
Phorate	104.13 ± 22.45	99.75 ± 0.11
Pirimicarb	11.67 ± 0.52	90.26 ± 0.33
Pirimiphos-ethyl	1.09 ± 0.08	95.16 ± 0.46
Propetamphos	> 120	91.32 ± 1.7
Pyrazophos	2.29 ± 0.71	88.65 ± 0.14
Quinalphos	2.49 ± 0.04	96.66 ± 0.83
Tebupirimfos	3.59 ± 0.08	94.85 ± 0.13
2,3,4,4 -Tetrahydroxybenzophenone	20.98 ± 0.65	83.27 ± 3.32
Terbufos	1.12 ± 0.2	98.65 ± 1.08
Thiobencarb	1.48 ± 0.54	98.6 ± 0.04
Triazophos	2.34 ± 0.25	86.23 ± 2.05
Tri-o-cresyl phosphate	1.17 ± 0.08	93.36 ± 0.84

The amount of the parent compound was measured by mass spectrometry at 0, 5, 10, 15, 30, and 60 min after microsome treatment. The half-lives were calculated based on the amount of the parent compounds at each time point. The amount of the compounds was measured after 0 and 16 h of the incubation with cells and microsomes. The depletion rates were calculated as % of the amount of the compounds at 0 h. Not found; the compounds were not detected in the MS spectrum. Not Metabolized; the parent compounds were stable in the assay condition.

### Comparison of metabolic capability between RLM and HLM by in silico metabolism prediction

**Fig. 4:**
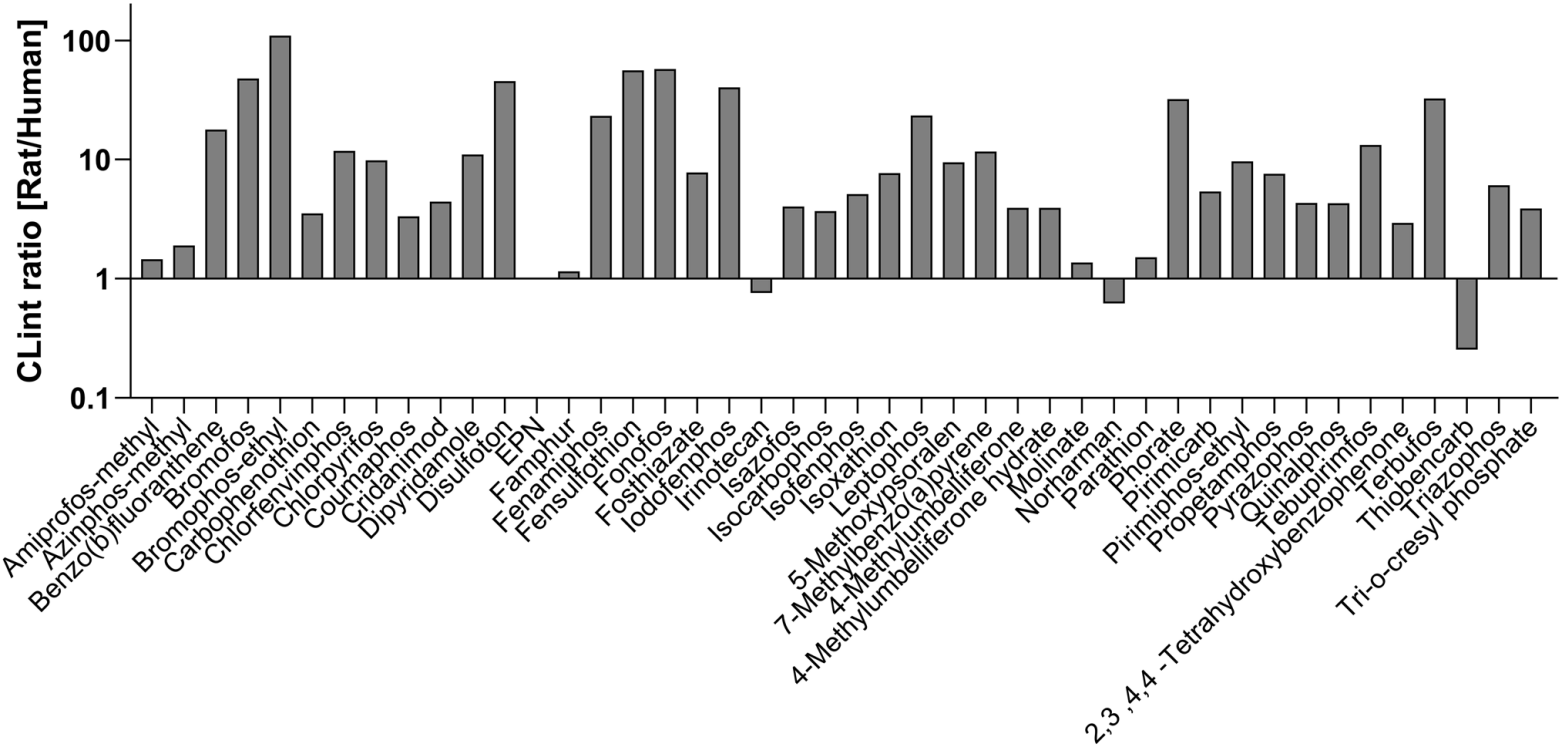
The ratio of the CL_int_ for rat and human liver microsomes. The CL_int_ was predicted using ADMET predictor. The y axis indicates the ratios that were calculated as rat microsomes CL_int_/human microsomes CL_int_

To investigate differences in metabolic capability between RLM and HLM, we compared microsomal activity using the in silico metabolism predictor, ADMET Predictor^®^. This software generates metabolism predictions based on experimental data. It provides the Michaelis–Menten constant (*K*_m_), maximum metabolic rate (*V*_max_), and intrinsic clearance (CL_int_) value, where CL_int_ was calculated by *V*_max_/*K*_m_. We compared CL_int_ between RLM and HLM for the 44 compounds tested for parent compound depletion in the presence of RLM. The results showed that RLM has much higher CL_int_ for most of the compounds compared to HLM, indicating that RLM is more effective at metabolizing these compounds than HLM (Fig. 4).

## Discussion

In this study, we applied an in vitro metabolism method to detect compounds requiring metabolic activation to induce a biological response. We used a cell-based p53-bla assay (CellSensor^™^ p53RE-bla HCT-116 cells) in a qHTS platform (Witt et al. 2017) to profile the Tox21 10K compound library in the presence and the absence of liver microsomes. SAR analysis was used to characterize the structural features of the identified p53 activators (Fig. 1). A subset of potential p53 activators was tested to confirm the requirement for metabolism using a high-throughput parent compounds depletion assay, followed by in silico predictive metabolism modeling. This approach proved to be an efficient method for screening a large chemical library to identify compounds that need metabolism to become active.

Among 278 selected compounds, 73 compounds demonstrated enhanced potency and/or efficacy when tested in the presence of RLM compared with the activity observed in the absence of RLM. Only 2 compounds showed higher p53 activation with HLM. These data suggest that RLM has higher metabolism capability, an observation supported by the comparison of CL_int_ values for RLM and HLM by ADMET Predictor^®^ (Fig. 4). These prediction data are supported by the previously reported experimental data comparing the metabolizing kinetics between induced RLM vs non-induced HLM by measuring the amount of the metabolites of known target compounds of major CYPs (Easterbrook et al. 2001). Although there are differences of opinion regarding the preferred species of microsomes to use in toxicology or pharmacology testing, and intuitively, use of HLM might be preferred, our data suggest that Aroclor 1254-induced RLM are more effective metabolizers than non-induced HLM, and therefore may be more effective for the purpose of hazard identification (Blais et al. 2017; Cox et al. 2016; Sakai et al. 2015).

Several compounds (e.g., etoposide) showed inactivity or lower activity (as measured by potency and/or efficacy) in p53 induction in the presence of microsomes compared with the activity levels observed in the absence of microsomes. We postulate that this reduction in bioactivity is most likely due to non-specific protein binding because similar results were observed with heat-attenuated microsomes (data not shown). Similar findings were also observed in our previously conducted screening for acetylcholinesterase inhibitors (Li et al. 2021).

Due to changes in product availability, different lots of HLM and RLM were used in the confirmation studies. As shown in Supplementary Figure S2, this new lot of HLM was markedly less effective at inducing biotransformation than the HLM used in the primary screening. This demonstrates one of the difficulties in using HLM, which is potential lot-to-lot variability due to different donors with different CYP profiles (Ahmad et al. 2018). Although a different lot of RLM was also used in the confirmation study, the results were similar to those from the primary study, and the p53-bla plus RLM assay showed high reproducibility. Despite the same trends in the primary screening and the confirmation study with RLM, aflatoxin B1 showed a tenfold higher potency in the confirmation study compared to the primary screening (Supplementary Table. S1). The activity of aflatoxin B1 may be modulated by a particular CYP subtype responsible for metabolism of the compound (Vleet et al. 2002). Different lots of microsomes may demonstrate slightly different CYP profiles, leading to changes in compound potency.

The Tox21 10K compound library was grouped into 1014 clusters based on structural similarity. Twenty-two clusters were enriched with p53 inducers from the p53-bla plus RLM assay, and 15 clusters were enriched with p53 inducers from the p53-bla plus HLM assay. Eleven clusters were associated with both species of microsomes; however, as shown in Fig. 2, most compounds showed higher potency when treated with RLM than with HLM. The enriched clusters mainly contain organophosphates, organothiophosphates, and organochlorines, chemical classes that are often used as pesticides. Results from an earlier study looking at inhibition of AChE activity in a cell-free system (Li et al. 2021) confirmed the need for many organophosphates, such as chlorpyrifos, to undergo biotransformation for activity. Enhanced activity following biotransformation is not limited to pesticides, but extends to a number of other environmental compounds such as polycyclic aromatic hydrocarbons (Sen et al. 2013).

Although 73 compounds showed higher potency and/or efficacy in inducing p53 signaling in the assay with RLM, 29 of these compounds did not show reduced activity in the p53-bla assay with heat-attenuated microsomes or in the absence of NADPH. The lack of reduced activity may be the result of the incomplete deactivation of microsomes by heat and the small amount of endogenous NADPH within cells, allowing some compounds to still undergo activation in our system. Some of the compounds showed higher potency with heat-attenuated microsomes than with fully functional microsomes (Supplementary Figure S3). One explanation for this observation is that the concentration–response curves for these compounds reached a plateau at low concentrations with heat attenuation or without NADPH, resulting in lower EC_50_ than from the assay with microsomes. Another possibility might be that heat attenuation and a low amount of NADPH slows down parent compound metabolism and intermediate, transient metabolites might have higher p53-inducing activity than the final metabolites.

The follow-up assays with heat-attenuated microsomes and no NADPH identified 44 compounds that require metabolism to induce p53 signaling. These 44 compounds were tested in the parent compound depletion assay. Despite the significantly higher p53-inducing activity after metabolic activation in the p53-bla assay, 5 compounds were unchanged in the parent compound depletion assay. One of these compounds was irinotecan, a pro-drug whose metabolite, SN-38, is known to be much more toxic than the parent compound (Ramesh et al. 2010). One possible explanation for the inability to detect depletion of irinotecan and the other 4 compounds is that the parent compound is relatively stable in the presence of microsomes, but a small amount of metabolite is sufficient to induce detectable p53 activation.

Current qHTS in vitro assays used in the Tox21 screening program lack metabolic capability, resulting in the failure to detect compounds whose metabolites may have bioactivity. This lack of metabolic capacity also does not allow identification of active parent compounds that are detoxified by liver microsomes. This limits the usefulness of these assays. Although a variety of in vitro metabolism methods, such as recombinant proteins, liver microsomes, and S9 liver fractions, have been explored in an HTS format, there remain several limitations in translating the data to human risk under real-life exposure conditions. One problem is that exogenous metabolism methods derived from induced rat livers (microsomes, S9 fraction) provide enzyme preparations that have higher metabolic capability than the human liver due to higher proportions of certain CYPs (Brandon et al. 2003). The metabolites produced by a rat metabolic system might not be the same as those produced from a human metabolic system. In addition, the liver microsome fraction only contains Phase 1 metabolic enzymes: it lacks phase II metabolic enzymes. Therefore, this method is not expected to identify the compounds that need phase II metabolism to induce a biological response. On the other hand, non-induced human-derived metabolism systems have less metabolic capability and large lot-to-lot variation, hindering the identification of active metabolites and preventing comparison of results across experiments and among laboratories (Ahmad et al. 2018; Green et al. 2017; Miksys et al. 2003). Moreover, these methods of providing exogenous metabolic activation do not fully represent human (or rat) physiological conditions due to the lack of a full complement of metabolic enzymes and the absence of a physiologically relevant microenvironment.

To address these problems, several novel three-dimensional (3D) in vitro model systems with inherent metabolic capability have been developed. These 3D cell culture systems, such as spheroid hepatocyte cultures and a variety of organoid models and more complex microphysiological systems, provide one possibility for model systems with fully functional endogenous metabolism. The 3D spheroid hepatocyte culture model has more metabolic capability than a traditional 2D culture model (Li et al. 2021; Ramaiahgari et al. 2017). As in a normal physiological condition, cells in this 3D model can differentiate and interact (Bell et al. 2018). While spheroids are mostly derived from transformed cancer cells, organoids are derived from normal adult organs or pluripotent stem cells (Takebe et al. 2019). Therefore, the organoids derived from differentiated stem cells represent a good in vitro model with enhanced in vivo relevance. Given that brain organoids have been used in an HTS platform recently (Durens et al. 2020), it is possible that liver organoids can also one day be utilized in an HTS format to provide a human physiological relevant metabolic system. These novel approaches could be used to further characterize compounds that are identified as having activity, or enhanced activity, in the presence of RLM in qHTS screens.

In summary, we have developed and optimized a p53-bla assay with exogenous metabolic activation provided by RLM and HLM. We identified 44 compounds, including two known and forty-two potentially novel, p53 activators that need metabolism to induce p53 signaling. We also found RLM activated more compounds than did HLM. Supporting evidence for the efficacy of RLM came from in silico metabolism predictions for the CL_int_ of each species of microsome. However, there may be inherent differences in the metabolites generated by RLM compared with metabolites generated by HLM, and these differences need to be explored and understood. Being able to generate human-relevant metabolizing systems for use in HTS formats remains a work in progress, but the development of such systems should be vigorously pursued. This study provides an example of applying an in vitro metabolism method to a qHTS format, and it may be of use in exploring other newly developed in vitro metabolism methods to a qHTS format. Ultimately, these efforts may provide more robust in vitro testing options to help meet the stated goal of the U.S. EPA to eliminate all animal testing by the year 2035 (Grimm 2020).

## Supplementary Information

Below is the link to the electronic supplementary material.

Supplementary file1 (DOCX 205 kb)

Supplementary file2 (XLSX 82 kb)

## Data Availability

The assay data are available at https://tripod.nih.gov/tox21/.

## Code availability

Metabolism prediction was run using ADMET predictor (https://www.simulations-plus.com).
