# Supplementary material for: Identification of environmental chemicals that activate p53 signaling after in vitro metabolic activation
Source: Arch Toxicol. 2022 Apr 18;96(7):1975–87. doi: 10.1007/s00204-022-03291-5 (PMC9151520; doi:10.1007/s00204-022-03291-5)
Supplement: Supplementary file 1 — Supplementary file1 (DOCX 205 kb) [file 204_2022_3291_MOESM1_ESM.docx]

Supplementary Figure. S1. Concentration–response curves of Aflatoxin B1 in p53RE assays. Cells were treated with Aflatoxin B1 in the presence of (a) human liver microsomes and (b) rat liver microsomes with different concentrations of NADPH. Y-axis, p53 response; X-axis, concentration of Aflatoxin B1.


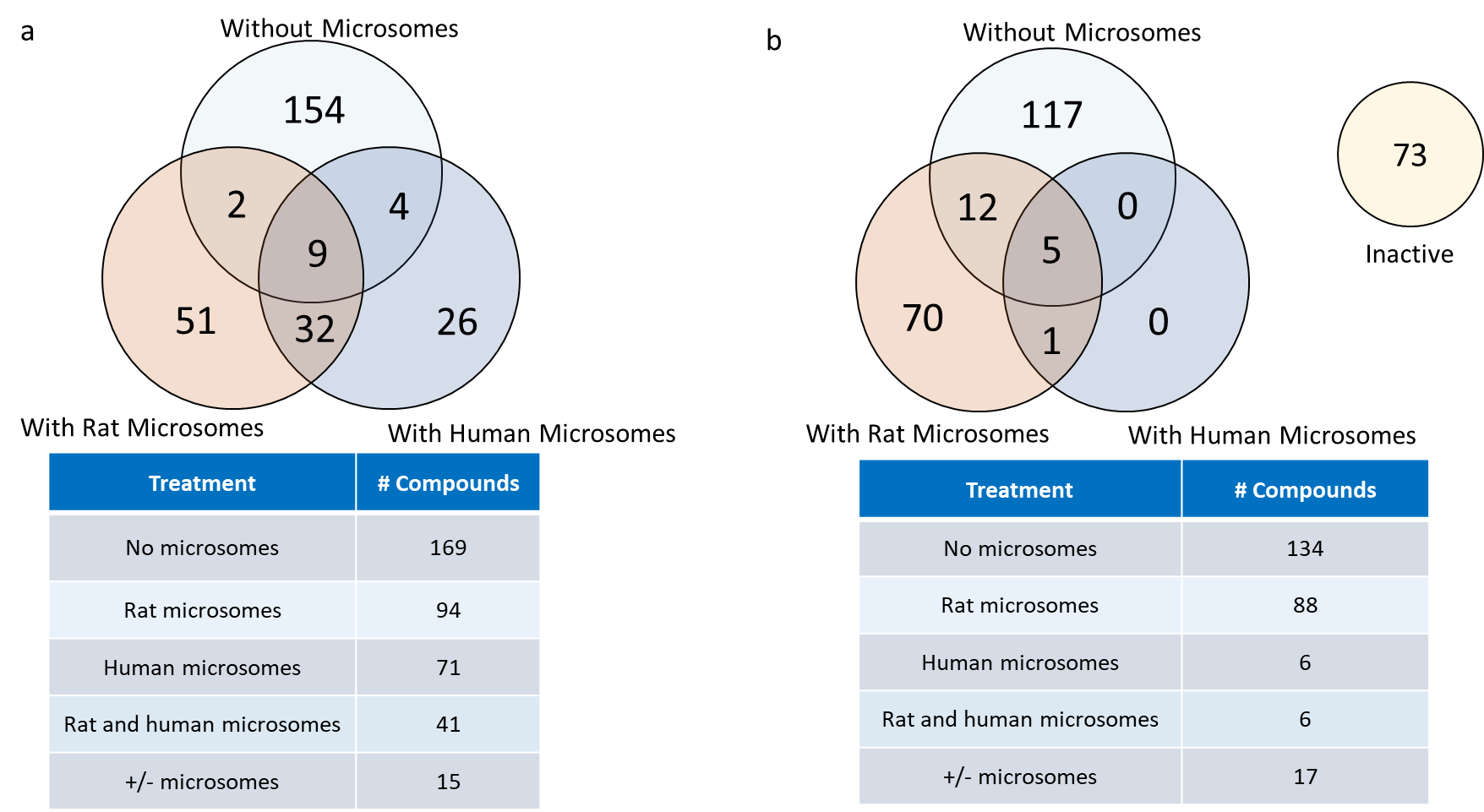


Supplementary Figure S2. Venn diagram showing the number of active compounds identified in (a) the primary screening and (b) the confirmation study under three conditions: without microsomes, with rat liver microsomes, or with human liver microsomes. The tables beneath each Venn diagram show the number of actives in each category.


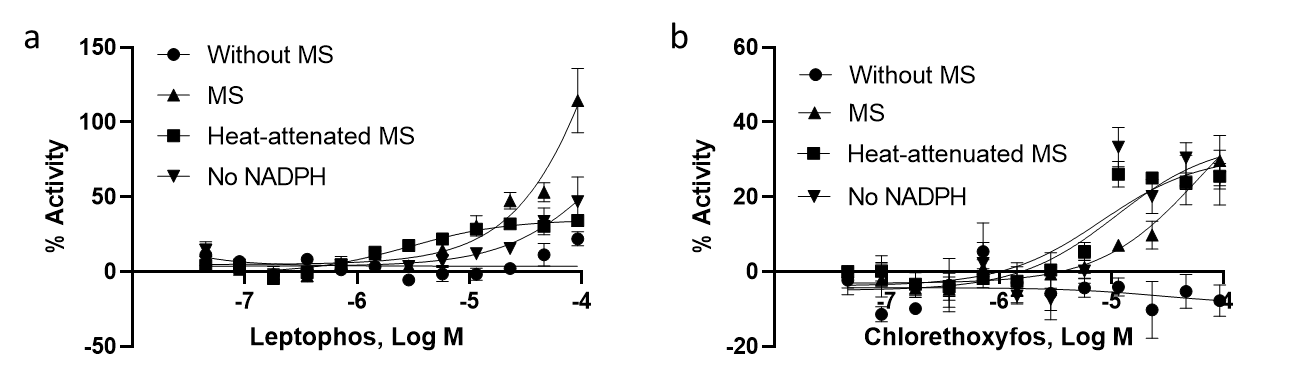


Supplementary Figure S3. Concentration–response curves of representative compounds in p53RE assays. Cells were treated with the compounds in the absence or presence of heat-attenuated rat liver microsomes or rat liver microsomes in the absence of NADPH. (a) leptophos, (b) chlorethoxyphos. Each value represents the mean ± SD of three independent experiments. MS, with microsomes; Heat attenuated MS, with heat attenuated microsomes; No NADPH, without NADPH.
